# County-Level Social Vulnerability, Metropolitan Status, and Availability of Home Health Services

**DOI:** 10.1001/jamanetworkopen.2023.37508

**Published:** 2023-10-13

**Authors:** Harriet Mather, Katherine A. Ornstein, Catherine McDonough, Bian Liu, Maggie Salinger, Christine S. Ritchie

**Affiliations:** 1 Brookdale Department of Geriatrics and Palliative Medicine, Icahn School of Medicine at Mount Sinai, New York, New York; 2 Johns Hopkins School of Nursing, Baltimore, Maryland; 3 currently a graduate student at Icahn School of Medicine at Mount Sinai, New York, New York; 4 Department of Population Health Science and Policy, Icahn School of Medicine at Mount Sinai, New York, New York; 5 Division of General Internal Medicine, Massachusetts General Hospital, Boston; 6 Mongan Institute Center for Aging and Serious Illness, Division of Palliative Care and Geriatric Medicine, Department of Medicine, Massachusetts General Hospital and Harvard Medical School, Boston

## Abstract

This cross-sectional study assesses county-level differences in home-based medical care and home health care by social vulnerability and metropolitan status.

## Introduction

Home-based medical care (HBMC) and skilled home health care (HHC) are key components of clinical care for the growing population of homebound older adults in the US.^1,2^ We examined differences in HBMC clinicians and skilled HHC agencies availability by area-level socioeconomic deprivation and metropolitan status across the US.

## Methods

This cross-sectional study followed the STROBE reporting guideline. Because publicly available data were used, institutional review board approval was not required in accordance with the Common Rule.

We generated county-level numbers of HBMC clinicians and HHC agencies per 1000 Medicare beneficiaries based on the zip codes of clinician address (from the 2019 Provider Utilization and Payment Data: Physician and Other Practitioners file) and patients served by that agency in the year (from the 2019 Home Health Care-Zip Codes file from the Care Compare: Home Health Quality Reporting Program), respectively. The number of Medicare beneficiaries was derived from the 2019 Medicare Data for Geographic Variation County-Level file. We used the 2020 Social Vulnerability Index (SVI) from the Centers for Disease Control and Prevention as a measure of county-level socioeconomic deprivation.^3^ Using 2013 Rural Urban Continuum Codes (RUCC), we classified counties as metropolitan (RUCC 1-3) and nonmetropolitan (RUCC 4-9).

We grouped counties into “low,” “middle,” and “high” SVI and HBMC and HHC density (including counties with no clinicians or agencies in the low tertile). We generated choropleth maps to covisualize county-level HBMC clinician and HHC agency densities and SVI. We calculated the proportion of nonmetropolitan counties in each of the resulting 9 matrix categories.

## Results

We included 3139 of 3144 counties in the analysis (5 had missing data on SVI or RUCC), with 1973 (62.9%) nonmetropolitan and 1166 (37.1%) metropolitan. Overall, 33 counties (1.1%) had no HHC agency or HBMC clinician (1765 [56.2%] had no HBMC, and 33 [1.1%] had no HHC agency). From the choropleth maps, we identified counties of high concern for underavailability of HBMC or HH (ie, low density of HBMC clinicians or HHC agencies and high SVI) (Figure); overall, 722 (23.0%) demonstrated underavailability of HBMC and 390 (12.4%) demonstrated underavailability of HHC. Nonmetropolitan counties were overrepresented among counties with low density of HBMC clinicians and underrepresented among counties with medium and high density of HBMC clinicians; in contrast, nonmetropolitan counties were overrepresented among counties with medium or high density of HHC agencies (Figure). These differences in HBMC and HHC density by metropolitan status were evident across all tertiles of SVI.

**Figure.**
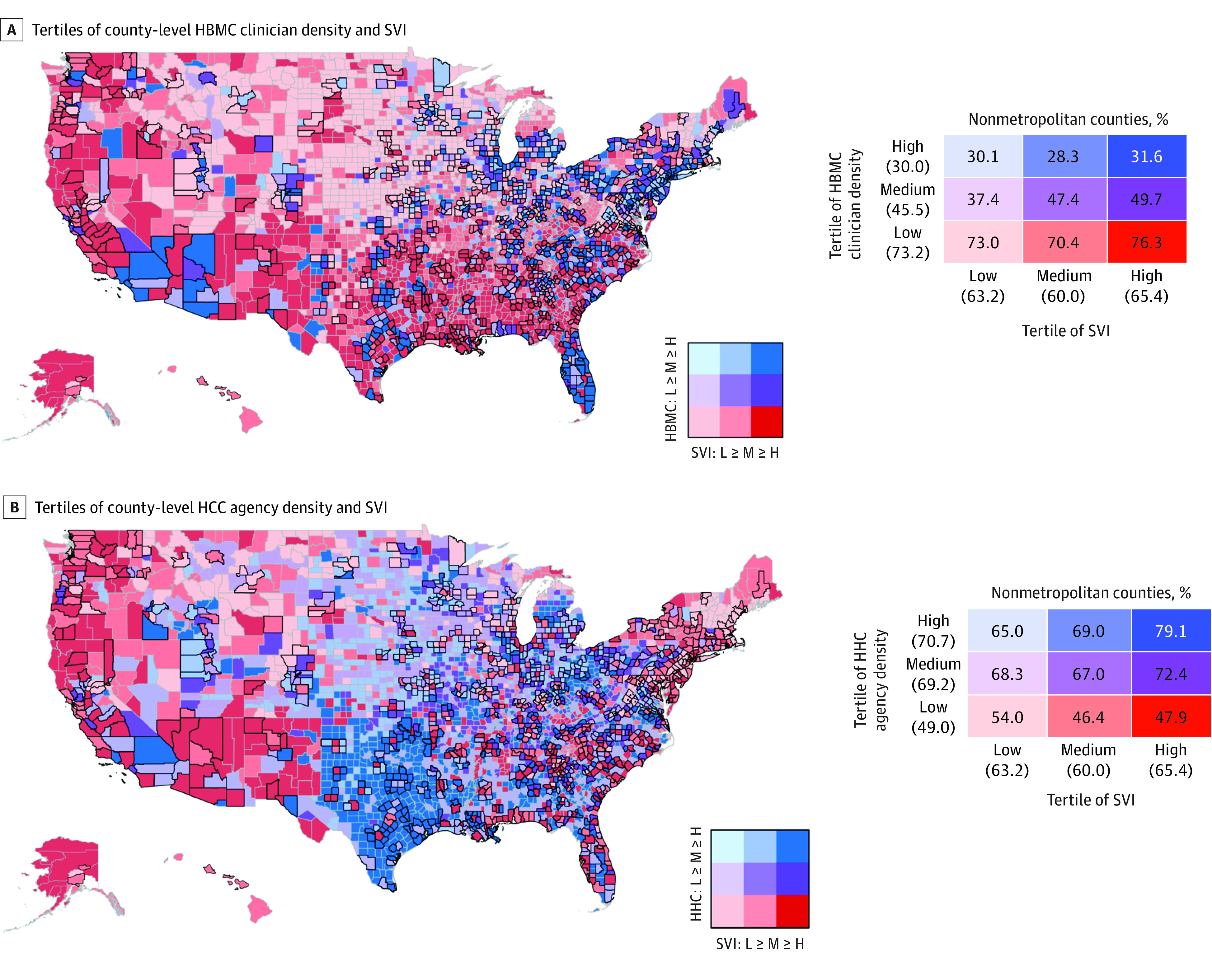
Density of Home-Based Medical Care (HBMC) Clinicians and Home Health Care (HHC) Agencies and Social Vulnerability Index (SVI) by Metropolitan Status Maps depict county-level density of HBMC clinicians (A) and HHC agencies (B) and social vulnerability in 9 bivariate categories. A thick border around a county indicates a metropolitan county. In the graphs to the right, the numbers in each of the 9 cells (and categories of SVI, HHC, and HBMC) represent the percentage of counties within that matrix category (and by category of SVI, HHC, and HBMC) that are nonmetropolitan. Tertiles of density of HBMC clinicians and HHC agencies are based on county-level numbers per 1000 Medicare beneficiaries. Tertiles of SVI are based on county-level SVI indexed to all counties in US. Counties with no HBMC clinicians (n = 1765) or HHC agencies (n = 33) were coded as low density. Absolute numbers of county-level HBMC clinicians per 1000 Medicare beneficiaries for each tertile are: 0.03 to 0.24 (low), 0.25 to 0.45 (medium), 0.46 to 4.31 (high). Absolute numbers of county-level HHC agencies per 1000 Medicare beneficiaries for each tertile are: 0.12 to 2.55 (low), 2.56 to 6.18 (medium), 6.19 to 570.1 (high). Overall, 1973 (62.9%) counties were nonmetropolitan and 1166 (37.1%) were metropolitan; 1626 of 2220 low-density (73.2%), 209 of 459 medium-density (49.0%), and 238 of 460 high-density (30.0%) HBMC counties were nonmetropolitan; and 522 of 1065 low-density (49.0%), 718 of 1037 medium-density (69.2%), and 744 of 1037 high-density (70.7%) HHC counties were nonmetropolitan. Metropolitan counties included counties with Rural-Urban Continuum Codes (RUCC) of 1 to 3, while nonmetropolitan counties had RUCCs of 4 to 9.

## Discussion

This cross-sectional study found differences in Medicare-funded home-based clinical care provision across the US by county-level SVI, suggesting inequitable care access among homebound Medicare beneficiaries; almost one-quarter of counties had low availability of HBMC clinicians coupled with high socioeconomic disadvantage. The relative lack of HBMC clinicians in nonmetropolitan areas was persistent at all levels of county-level socioeconomic disadvantage and may reflect lack of effective reimbursements to support provision of HBMC across large driving distances; expansion of telemedicine may serve to support access to home-based primary care in rural areas.^4^ We did not observe similar differences in HHC agency density by metropolitan status.

Our analysis was restricted to Medicare-funded home-based clinical care; while we included measures of density, we were unable to capture the capacity and quality of each clinician or agency. Further work is needed to establish the mechanisms, impact, and solutions to the county-level inequities of HBMC observed in this study and to identify the optimal level of HBMC and HHC provision for meeting the needs of clinically and socioeconomically vulnerable populations.

## Notes

Supplement.
Data Sharing Statement
