# Supplementary material for: County-Level Social Vulnerability, Metropolitan Status, and Availability of Home Health Services
Source: JAMA Netw Open. 2023 Oct 13;6(10):e2337508. doi: 10.1001/jamanetworkopen.2023.37508 (PMC10576214; doi:10.1001/jamanetworkopen.2023.37508)
Supplement: Supplement. — Data Sharing Statement [file jamanetwopen-e2337508-s001.pdf]

Data Sharing Statement

Mather. County-Level Social Vulnerability, Metropolitan Status, and Availability of Home Health Services. *JAMA Netw Open*. Published October 13, 2023.
doi:10.1001/jamanetworkopen.2023.37508

Data
Data available: No

Additional Information
Explanation for why data not available: The data are publicly available.
